# Disease burden comparison and associated risk factors of early- and late-onset neonatal sepsis in China and the USA, 1990–2019

**DOI:** 10.1080/16549716.2024.2396734

**Published:** 2024-09-04

**Authors:** Chengyue Zhang, Lianfang Yu, Xiaoming Pan, Yuwei Lu, Kaiyu Pan

**Affiliations:** a Department of Neurology, The Children’s Hospital, Zhejiang University School of Medicine, National Clinical Research Center for Child Health, Hangzhou, China; b Department of Pediatrics, Xiaoshan Affiliated Hospital of Wenzhou Medical University, Hangzhou, Zhejiang, China; c Department of Pediatrics, Hangzhou Ninth People’s Hospital, Hangzhou, Zhejiang, China; d Department of Pediatrics, Wenzhou Medical University, Wenzhou, Zhejiang, China

**Keywords:** Disability-adjusted life years, global burden of disease, low birth-weight, neonatal sepsis, short gestation, summary exposure value

## Abstract

**Background:**

The morbidity and mortality rates of neonatal sepsis are high, with significant differences in risk factors and disease burden observed between developing and developed countries.

**Objective:**

To provide evidence to support recommendations on improving public health policies using a comparative systematic analysis of the disease burden.

**Methods:**

Using data from the Global Burden of Disease Study 2019, the prevalence and incidence of early- and late-onset neonatal sepsis and the disability-adjusted life years (DALYs) due to both countries in both China and the United States of America (USA) were assessed. Furthermore, the DALYs and summary exposure values for the primary risk factors (short gestation and low birthweight) were analysed. Joinpoint regression models were used to analyse temporal trends in epidemiological indicators of neonatal sepsis.

**Results:**

Between 1990 and 2019, the incidence and prevalence of neonatal sepsis demonstrated a significant upwards trend in China, whereas both were largely stable in the USA. A decreasing trend in the DALYs due to neonatal sepsis caused by short gestation and low birthweight in both sexes was observed in both countries, whereas a fluctuating increasing trend in years lived with disability was observed in China.

**Conclusions:**

The aim of the Chinese public health policy should be to control risk factors, learning from the advanced health policy planning and perinatal management experiences of developed countries.

## Paper Context

**Main findings:**

Disability-adjusted life years (DALYs) attributed to short gestation and low birth-weight for neonatal sepsis have been decreasing in both China and the USA; years lived with disability (YLDs) and summary exposure values (SEVs) have been increasing in China.

**Added knowledge:**

This study provides new knowledge about the disease burden of neonatal sepsis attributable to short gestation and low birthweight and suggests possible interventions.

**Global health impact for policy and action:**

Public health policies in developing countries need to focus on moderating risk factors, learning from the advanced health policy planning and perinatal management experiences of developed countries, and improving neonatal follow-up and rehabilitation interventions.

## Background

Neonates, defined as infants aged <28 days (0–27 days) according to the Global Burden of Disease Study (GBD) 2019, are highly susceptible to neonatal sepsis, which accounts for 50% of deaths in children aged <5 years [1]. Neonatal sepsis is a condition characterised by the clinical signs of systemic infections caused by bacteria, fungi, or viruses, with bacteria being the most common pathogens [2]. In the present study, ‘neonatal sepsis’ refers to bloodstream infections occurring systematically in humans during the neonatal period. This condition can be classified as early- or late-onset, depending on the time of sepsis onset. According to the GBD 2019, early-onset neonatal sepsis is defined as sepsis occurring in early neonates aged 0–6 days, whereas late-onset neonatal sepsis occurs in older neonates aged 7–27 days [3]. Sepsis during the neonatal period is associated with significantly higher morbidity and mortality rate than does sepsis in any other stage of life [4]. The prevalence of sepsis ranges from 1 to 5 and 49 to 170 cases per 1000 live births in developed and developing countries, respectively [5]. In a study in which the causes of neonatal death in 194 countries was investigated, sepsis accounted for 15% of the total mortality rate [6]. Another survey in developing countries revealed that sepsis accounted for 40% of neonatal deaths [7].

Following advances in neonatology and the increased survival of infants with low birthweight, particularly those with very low birthweight, the incidence of mortality due to neonatal sepsis has decreased. However, this increased survival has been accompanied by long-term disability due to neurological complications in surviving infants [8,9]. Newborns with sepsis have an increased risk of developing periventricular leukomalacia, intraventricular haemorrhage, and respiratory distress syndrome compared with those without sepsis. Furthermore, survivors have an increased risk of long-term adverse outcomes, being twice as likely to develop cerebral palsy, as well as an increased risk of developing cognitive deficits, psychomotor retardation, and visual and auditory impairments [10].

The most critical risk factors for the development of neonatal sepsis are short gestation period and low birthweight [11]. Despite a decrease in the incidence of early-onset neonatal sepsis following improvements in obstetric care and increases in the use of intrapartum antibiotics [12], the incidence of late-onset neonatal sepsis has increased due to the increasing survival of preterm infants and those with very low birthweight [13]. As these infants are at a high risk of late-onset sepsis, an overall increase in the incidence of late-onset sepsis has been observed in these populations.

The incidence of neonatal sepsis may reflect differences in income levels among countries, disparities in healthcare resources and settings, and advances in medical technologies [14].

Neonatal sepsis is the most treatable and preventable cause of childhood infectious diseases globally [15]. However, despite its prevalence, no systematic comparative analysis of the morbidity, prevalence, or disease burden of neonatal sepsis between developing and developed countries has been reported in recent years.

China, classified as a ‘developing country’, reported a decrease in the mortality rate of neonatal sepsis from 0.4 to 0.1 per 1000 live births between 1996 and 2015 [16,17]. The United States of America (USA), a high-income country, reported a decrease in the in-hospital mortality rate of infants with neonatal sepsis from 4% in 2002 to 2% in 2014 owing to improvements in maternal and neonatal health care [18–20]. Significant differences in the mortality rates of neonatal sepsis are largely due to variations in economic development [21]. Therefore, the aim of the present study was to estimate and compare the prevalence and incidence of neonatal sepsis, disability-adjusted life years (DALYs) due to neonatal sepsis, and primary risk factors for neonatal sepsis between China and the USA over a 20-year period (1990–2019) using the GBD 2019 data. The study sought to provide evidence to inform measures that could be adopted to improve health policy planning and perinatal management in developing countries.

## Materials and methods

### Ethics statements

This study was conducted in accordance with the Guidelines for Accurate and Transparent Health Estimates Reporting [22]. Obtaining informed consent from participants was not required.

### Data source and study population

The Cause of Death Ensemble Model (CODEm) and spatiotemporal Gaussian process regression models were used in the GBD 2019 to estimate the disease burden using epidemiological indicators, such as the incidence and prevalence of 369 diseases and injuries in 204 countries and territories, and several comprehensive indicators such as DALYs, including years of life lost (YLLs) and years lived with disability (YLDs), with 87 attributable risk factors. Details on the specific methodology used to calculate the disease burden have been previously reported [3]. In addition, public information is available online (http://ghdx.healthdata.org/gbd-results-tool).

Data regarding neonatal sepsis according to nation, sex, and age between 1990 and 2019 was collected in the present study between 16 February and 18 February 2022. The following information on early- and late-onset neonatal sepsis cases among males and females in China and the USA was further collected: (1) incidence, prevalence, and mortality rates; (2) DALYs, YLLs, and YLDs; (3) all risk factors for neonatal sepsis in the aforementioned population in the GBD 2019 and the summary exposure values (SEVs) for low birthweight and short gestation [23]. According to data from the World Bank database, China’s adjusted net national income per capita in 2021 was $9,015 compared with $59,009 in the USA [24].

In the GBD 2019, neonatal sepsis was defined according to the International Consensus Conference on Pediatric Sepsis Definitions, American College of Chest Physicians/Society of Critical Care Medicine Consensus Criteria, or sepsis-relevant International Classification of Diseases (ICD)-9/ICD-10 codes or code combinations, as it is clinically diagnosed and laboratory-confirmed based on positive blood cultures [1,25]. DALYs were calculated as the sum of years of life lost due to premature death (YLLs) and nonfatal health impairment (YLDs) according to cause, age, and sex [26]. Short gestation was defined as a gestational age at birth of less than the lowest-risk age (38 weeks), whereas low birthweight was defined as a birthweight less than the lowest-risk weight (2500 g). Both factors are leading level 4 risk factors for diseases in children aged <5 years [27]. The SEV represents the exposure dose of a population to a risk factor, resulting from excess risk-weighted prevalence [28]. SEV values range from zero to one hundred, with zero representing no risk of exposure to the population and one hundred representing the highest risk level of exposure [29].

### Statistical analysis

To determine the trends in different epidemiological indicators over time, the Joinpoint Regression Program (version 4.9.0.0; National Cancer Institute, Rockville, MD, USA) was used. This programme identifies statistically significant trend segments by establishing interval segmentation function joinpoints with minimum mean squared errors and the interval function fit following the sequential testing procedure [30]. To ensure the credibility of the results, the maximum number of joinpoints was 3, and the annual percentage change (APC), average annual percentage change (AAPC), and 95% confidence interval (CI) for the different stages were subsequently derived. The AAPC was calculated as the average weighted by the length of the APC interval, and the Z test was applied to determine if it was significantly different from zero. Statistical significance was set at *p* < 0.05. All epidemiological indicators are expressed in units of 100,000 live births, except for SEVs, which are expressed as percentages. All estimates were age-standardised.

## Results

### Temporal trends in the prevalence and incidence of neonatal sepsis, 1990–2019

**Figure 1.**
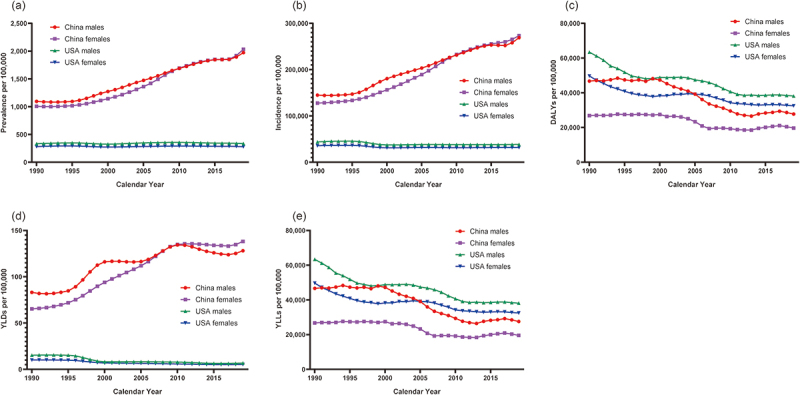
The temporal trends in the age-standardized prevalence, incidence, DALYs, YLDs and YLLs per 100,000 population of neonates with early-onset neonatal sepsis by sex in China and the USA, 1990–2019. (a). prevalence. (b). incidence. (c). DALYs. (d). YLDs. (e). YLLs.

Abbreviation: DALYs disability-adjusted life years; YLDs years lived with disability; YLLs years of life lost

**Figure 2.**
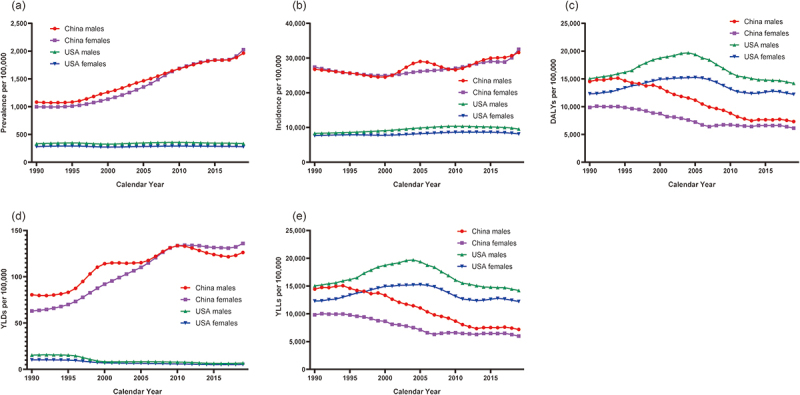
The temporal trends in the age-standardized prevalence, incidence, DALYs, YLDs and YLLs per 100,000 population of neonates with late-onset neonatal sepsis by sex in China and the USA, 1990–2019. (a). prevalence. (b). incidence. (c). DALYs. (d). YLDs. (e). YLLs.

Abbreviation: DALYs disability-adjusted life years; YLDs years lived with disability; YLLs years of life lost

The temporal trends in the prevalence and incidence of early- and late-onset neonatal sepsis according to sex in China and the USA are presented in Figure 1 (a, b) and Figure 2 (a, b), respectively, whereas the respective specific APC values are presented in Supplementary Table S1 and Supplementary Table S2. Details regarding the values of each epidemiological indicator rate in 1990 and 2019 are presented in Supplementary Table S3. Compared with the prevalence of neonatal sepsis in the USA, which remained roughly constant throughout the study period, the prevalence in China exhibited an upward trend, indicating a significant increase in the proportion of neonates with early-onset (APC: 2.6; 95% CI: 0.9, 4.2) and late-onset (APC: 2.6; 95% CI: 1.0, 4.3) neonatal sepsis, particularly between 2017 and 2019. Among the neonates with early-onset neonatal sepsis, an overall decreasing trend in the incidence of neonatal sepsis was observed in the USA, whereas a significant overall increasing trend was observed in China. The incidence of late-onset neonatal sepsis increased in both the USA and China over the study period, with the increasing trend being higher among neonates in China (males: AAPC, 0.6; 95% CI, 0.3, 0.8; females: AAPC, 0.6; 95% CI, 0.4, 0.7) than among neonates in the USA (males: AAPC, 0.5; 95% CI, 0.4, 0.6; females: AAPC, 0.2; 95% CI, 0.0, 0.3). In addition, when comparing neonates with early- and late-onset neonatal sepsis, a higher incidence of neonatal sepsis was observed in neonates with early-stage neonatal sepsis than in those with late-stage neonatal sepsis in China in 2019.

### Temporal trends in the DALYs, YLDs, and YLLs due to neonatal sepsis, 1990–2019

The temporal trends in the DALYs, YLDs, and YLLs due to early- and late-onset neonatal sepsis stratified by sex in China and the USA are presented in Figure 1 (C, D, E) and Figure 2 (C, D, E), respectively, whereas the exact values are detailed in the joinpoint regression models present in Tables 1 and 2. A relatively significant decreasing trend in the DALYs due to early-onset neonatal sepsis was observed in both countries. However, a consistent and overall decreasing trend in the DALYs due to late-onset neonatal sepsis was observed in China compared with that in the USA, where the DALYs initially increased and subsequently decreased (starting in 2003 for male neonates and 2006 for female neonates). As of 2019, the DALYs for both sexes were higher in the USA than that in China, with male neonates and neonates with early-onset neonatal sepsis having higher DALYs than those of female neonates and neonates with late-onset neonatal sepsis, respectively, in both countries. Regarding YLDs due to neonatal sepsis, a fluctuating significant increasing trend was observed in both male (AAPC: 1.4; 95% CI: 0.9, 2.0) and female (AAPC: 2.6; 95% CI: 2.3, 2.8) neonates in China, whereas an overall decreasing trend was observed in both male (AAPC: −3.1; 95% CI: −3.9, −2.3) and female neonates (AAPC: −2.3; 95% CI: −2.8, −1.8) neonates in the USA. No significant differences in the change in the curve were observed between neonates with early- and late-onset neonatal sepsis. The trends in YLLs and DALYs were the same in both countries.

**Table 1.** Trends in DALYs, YLDs and YLLs of early-onset neonatal sepsis by sex in China and the USA, 1990 - 2019, using joinpoint regression models.

	DALYs		YLDs		YLLs		DALYs due to short gestation		DALYs due to low birthweight	
	Time interval	APC (95% CI)	Time interval	APC (95% CI)	Time interval	APC (95% CI)	Time interval	APC (95% CI)	Time interval	APC (95% CI)
China males										
Trend 1	1990-2000	0.3 (−0.1, 0.6)	1990-1995	0.4 (−1.1, 1.9)	1990-2000	0.3 (−0.1, 0.6)	1990-2000	−0.2 (−0.6, 0.1)	1990-2000	−0.6 (−1.0, −0.2) *
Trend 2	2000-2012	−4.8 (−5.1, −4.5)*	1995-1999	7.5 (4.0, 11.1)*	2000-2012	−4.8 (−5.1, −4.5)*	2000-2012	−4.4 (−4.7, −4.1)*	2000-2012	−5.0 (−5.4, −4.7)*
Trend 3	2012-2017	1.8 (0.3, 3.4)*	1999-2011	1.4 (0.9, 1.9)*	2012-2017	1.8 (0.3, 3.4)*	2012-2017	3.2 (1.7, 4.7)*	2012-2017	2.7 (1.1, 4.2)*
Trend 4	2017-2019	−2.7 (−7.2, 2.1)	2011-2019	−0.8 (−1.5, −0.0)*	2017-2019	−2.7 (−7.3, 2.1)	2017-2019	−1.2 (−5.6, 3.4)	2017-2019	−1.8 (−6.4, 3.0)
AAPC	1990-2019	−1.8 (−2.2, −1.4)*	1990-2019	1.4 (0.9, 2.0)*	1990-2019	−1.8 (−2.2, −1.4)*	1990-2019	−1.5 (−1.9, −1.1)*	1990-2019	−2.0 (−2.4, −1.6)*
China females										
Trend 1	1990-2003	−0.1 (−0.5, 0.2)	1990-1995	1.9 (1.3, 2.5)*	1990-2003	−0.1 (−0.5, 0.2)	1990-2003	−0.4 (−0.7, −0.0)*	1990-2003	−0.9 (−1.2, −0.5)*
Trend 2	2003-2007	−7.5 (−10.7, −4.3)*	1995-1999	6.1 (4.8, 7.5)*	2003-2007	−7.6 (−10.8, −4.3)*	2003-2007	−6.9 (−10.0, −3.6)*	2003-2007	−7.6 (−10.6, −4.4)*
Trend 3	2007-2012	−0.8 (−3.0, 1.4)	1999-2010	3.8 (3.6, 4.0)*	2007-2012	−0.8 (−3.0, 1.4)	2007-2012	−0.1 (−2.2, 2.1)	2007-2012	−0.5 (−2.6, 1.6)
Trend 4	2012-2019	1.4 (0.5, 2.3)*	2010-2019	−0.1 (−0.3, 0.2)	2012-2019	1.4 (0.5, 2.4)*	2012-2019	2.4 (1.5, 3.4)*	2012-2019	1.9 (1.0, 2.8)*
AAPC	1990-2019	−0.9 (−1.6, −0.3)*	1990-2019	2.6 (2.3, 2.8)*	1990-2019	−1.0 (−1.6, −0.3)*	1990-2019	−0.6 (−1.2, 0.0)	1990-2019	−1.1 (−1.7, −0.5)*
USA males										
Trend 1	1990-1996	−4.2 (−4.6, −3.8)*	1990-1996	−0.9 (−2.4, 0.6)	1990-1997	−4.2 (−4.6, −3.8)*	1990-1997	−3.4 (−3.6, −3.2)*	1990-1996	−3.8 (−4.1, −3.4)*
Trend 2	1996-2006	−0.3 (−0.5, −0.1) *	1996-2000	−14.5 (−18.3, −10.6)*	1997-2006	−0.3 (−0.5, −0.1)*	1997-2005	0.4 (0.2, 0.7)*	1996-2005	0.2 (0.0, 0.5)*
Trend 3	2006-2011	−4.0 (−4.7, −3.3)*	2000-2005	1.1 (−1.7, 4.0)	2006-2011	−4.0 (−4.7, −3.3)*	2005-2012	−3.5 (−3.8, −3.2)*	2005-2012	−3.3 (−3.6, −2.9)*
Trend 4	2011-2019	−0.2 (−0.5, 0.1)	2005-2019	−2.0 (−2.4, −1.6)*	2011-2019	−0.2 (−0.5, 0.1)	2012-2019	−0.0 (−0.3, 0.2)	2012-2019	0.1 (−0.2, 0.4)
AAPC	1990-2019	−1.7 (−1.9, −1.6) *	1990-2019	−3.1 (−3.9, −2.3)*	1990-2019	−1.7 (−1.9, −1.6)*	1990-2019	−1.6 (−1.7, −1.4)*	1990-2019	−1.5 (−1.6, −1.3)*
USA females										
Trend 1	1990-1997	−3.5 (−3.8, −3.2)*	1990-1996	−1.0 (−1.7, −0.2)*	1990-1997	−3.5 (−3.8, −3.2)*	1990-1997	−3.2 (−3.5, −2.9)*	1990-1997	−3.1 (−3.4, −2.8)*
Trend 2	1997-2006	0.3 (0.1, 0.6)*	1996-1999	−9.5 (−13.5, −5.3)*	1997-2006	0.3 (0.1, 0.6)*	1997-2006	0.7 (0.4, 0.9)*	1997-2006	0.7 (0.5, 1.0)*
Trend 3	2006-2011	−3.1 (−3.8, −2.5)*	1999-2017	−2.0 (−2.2, −1.8)*	2006-2011	−3.1 (−3.8, −2.5)*	2006-2011	−3.4 (−4.1, −2.7)*	2006-2011	−3.2 (−3.9, −2.5)*
Trend 4	2011-2019	−0.3 (−0.6, −0.1)*	2017-2019	2.3 (−2.2, 7.1)	2011-2019	−0.3 (−0.6, −0.1)*	2011-2019	−0.4 (−0.6, −0.1)*	2011-2019	−0.2 (−0.5, 0.0)
AAPC	1990-2019	−1.4 (−1.6, −1.2)*	1990-2019	−2.3 (−2.8, −1.8)*	1990-2019	−1.4 (−1.5, −1.2)*	1990-2019	−1.3 (−1.4, −1.1)*	1990-2019	−1.2 (−1.3, −1.0)*

*Significantly different from 0 (*p* < 0.05).

*Abbreviation*: DALYs disability-adjusted life years; YLDs years lived with disability; YLLs years of life lost; APC annual percent change; AAPC average annual percent change; CI confidential interval.

**Table 2.** Trends in DALYs, YLDs and YLLs of late-onset neonatal sepsis by sex in China and the USA, 1990 - 2019, using joinpoint regression models.

	DALYs		YLDs		YLLs		DALYs due to short gestation		DALYs due to low birthweight	
	Time interval	APC (95% CI)	Time interval	APC (95% CI)	Time interval	APC (95% CI)	Time interval	APC (95% CI)	Time interval	APC (95% CI)
China males										
Trend 1	1990-1994	0.8 (−0.7, 2.3)	1990-1995	0.6 (−0.9, 2.1)	1990-1994	0.8 (−0.7, 2.3)	1990-1994	0.6 (−0.8, 2.1)	1990-1994	0.4 (−1.0, 1.8)
Trend 2	1994-2001	−2.0 (−2.8, −1.3)*	1995-1999	7.5 (4.0, 11.2)*	1994-2001	−2.1 (−2.9, −1.3)*	1994-2000	−2.2 (−3.2, −1.2)*	1994-2000	−2.6 (−3.6, −1.7)*
Trend 3	2001-2013	−4.4 (−4.7, −4.1)*	1999-2011	1.5 (1.0, 2.0)*	2001-2013	−4.4 (−4.7, −4.1)*	2000-2013	−3.4 (−3.7, −3.2)*	2000-2013	−4.3 (−4.5, −4.0)*
Trend 4	2013-2019	−0.3 (−1.1, 0.5)	2011-2019	−0.9 (−1.7, −0.2)*	2013-2019	−0.3 (−1.1, 0.5)	2013-2019	1.1 (0.3, 1.8)*	2013-2019	0.4 (−0.3, 1.2)
AAPC	1990-2019	−2.3 (−2.6, −2.0)*	1990-2019	1.5 (0.9, 2.0)*	1990-2019	−2.3 (−2.6, −2.0)*	1990-2019	−1.7 (−2.0, −1.4)*	1990-2019	−2.3 (−2.7, −2.0)*
China females										
Trend 1	1990-1997	−0.6 (−1.2, 0.0)	1990-1995	2.1 (1.4, 2.7)*	1990-1997	−0.6 (−1.3, 0.0)	1990-1995	−0.3 (−1.2, 0.7)	1990-1996	−1.0 (−1.8, −0.3)*
Trend 2	1997-2007	−3.7 (−4.1, −3.3)*	1995-1999	6.2 (4.7, 7.7)*	1997-2007	−3.8 (−4.3, −3.4)*	1995-2007	−3.0 (−3.3, −2.8)*	1996-2007	−3.9 (−4.2, −3.6)*
Trend 3	2007-2017	−0.1 (−0.5, 0.4)	1999-2010	3.9 (3.7, 4.1)*	2007-2017	−0.1 (−0.5, 0.4)	2007-2017	1.0 (0.6, 1.4)*	2007-2017	0.3 (−0.1, 0.7)
Trend 4	2017-2019	−3.3 (−7.9, 1.4)	2010-2019	−0.2 (−0.5, 0.0)	2017-2019	−3.4 (−8.0, 1.5)	2017-2019	−2.7 (−6.8, 1.6)	2017-2019	−3.2 (−7.3, 1.1)
AAPC	1990-2019	−1.7 (−2.1, −1.3)*	1990-2019	2.6 (2.4, 2.8)*	1990-2019	−1.8 (−2.1, −1.4)*	1990-2019	−1.2 (−1.5, −0.8)*	1990-2019	−1.8 (−2.2, −1.5)*
USA males										
Trend 1	1990-2003	2.3 (2.1, 2.5)*	1990-1996	−0.9 (−2.4, 0.7)	1990-2003	2.3 (2.1, 2.5)*	1990-1993	1.8 (0.2, 3.5)*	1990-2003	2.8 (2.6, 3.0)*
Trend 2	2003-2007	−1.8 (−3.5, 0.0)	1996-2000	−14.4 (−18.2, −10.4)*	2003-2007	−1.8 (−3.5, 0.0)	1993-2004	2.9 (2.6, 3.1)*	2003-2007	−1.5 (3.4, 0.4)
Trend 3	2007-2011	−4.4 (−6.1, −2.7)*	2000-2006	0.7 (−1.4, 2.7)	2007-2011	−4.4 (−6.2, −2.7)*	2004-2012	−3.6 (−4.1, −3.2)*	2007-2011	−4.5 (−6.3, −2.7)*
Trend 4	2011-2019	−1.0 (−1.3, −0.6)*	2006-2019	−2.2 (−2.7, −1.7)*	2011-2019	−1.0 (−1.3, −0.6)*	2012-2019	−0.9 (−1.4, −0.5)*	2011-2019	−0.9 (−1.3, −0.5)*
AAPC	1990-2019	−0.1 (−0.5, 0.2)	1990-2019	−3.1 (−3.9, −2.4)*	1990-2019	−0.1 (−0.5, 0.3)	1990-2019	0.0 (−0.2, 0.2)	1990-2019	0.1 (−0.2, 0.5)
USA females										
Trend 1	1990-2000	2.1 (1.9, 2.4)*	1990-1996	−1.0 (−1.8, −0.2)*	1990-2000	2.1 (1.9, 2.4)*	1990-2001	2.5 (2.3, 2.8)*	1990-2000	2.6 (2.3, 2.8)*
Trend 2	2000-2006	0.5 (−0.2, 1.2)	1996-1999	−9.3 (−13.8, −4.7)*	2000-2006	0.5 (−0.2, 1.2)	2001-2006	0.6 (−0.4, 1.6)	2000-2006	0.9 (0.2, 1.6)*
Trend 3	2006-2011	−3.8 (−4.8, −2.8)*	1999-2017	−2.1 (−2.2, −1.9)*	2006-2011	−3.8 (−4.8, −2.8)*	2006-2012	−3.6 (−4.3, −2.9)*	2006-2011	−3.9 (−4.8, −3.0)*
Trend 4	2011-2019	−0.2 (−0.6, 0.1)	2017-2019	2.2 (−2.8, 7.4)	2011-2019	−0.2 (−0.6, 0.1)	2012-2019	−0.0 (−0.5, 0.4)	2011-2019	−0.2 (−0.5, 0.2)
AAPC	1990-2019	0.1 (−0.1, 0.3)	1990-2019	−2.3 (−2.9, −1.7)*	1990-2019	0.1 (−0.1, 0.3)	1990-2019	0.3 (0.0, 0.5)*	1990-2019	0.3 (0.1, 0.6)*

*Significantly different from 0 (*p* < 0.05).

*Abbreviation*: DALYs disability-adjusted life years; YLDs years lived with disability; YLLs years of life lost; APC annual percent change; AAPC average annual percent change; CI confidential interval.

### Temporal trends in DALYs due to short gestation and low birthweight, 1990–2019

**Figure 3.**
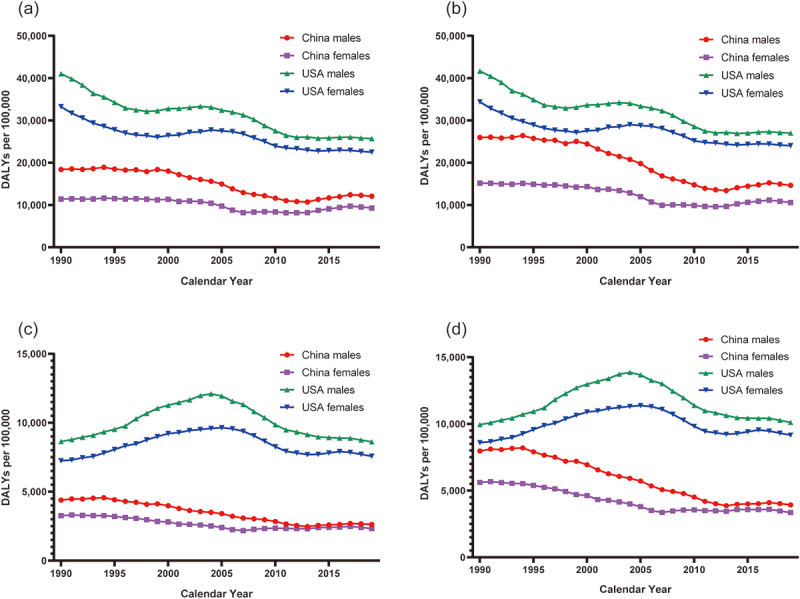
The temporal trends in the age-standardized DALYs per 100,000 population of early- and late-onset neonatal sepsis due to short gestation and low birth weight by sex in China and the USA, 1990–2019. (a). DALYs due to short gestation in neonates with early-onset neonatal sepsis. (b). DALYs due to low birth weight in neonates with early-onset neonatal sepsis. (c). DALYs due to short gestation in neonates with late-onset neonatal sepsis. (d). DALYs due to low birth weight in neonates with late-onset neonatal sepsis.

Abbreviation: DALYs disability-adjusted life years

The curves of DALYs per year for neonates with neonatal sepsis caused by short gestation and low birthweight are shown in Figure 3 and are supported by the joinpoint regression models presented in Tables 1 and 2. Overall, a significant downward trend in the DALYs due to early-onset neonatal sepsis caused by short gestation and low birthweight was observed between 1990 and 2019 in both the USA and China. Furthermore, the DALYs for both sexes were lower in China than in the USA. Regarding this decreasing trend, male neonates with early-stage neonatal sepsis in China had the highest rate of change (AAPC: −2.0; 95% CI: −2.4, −1.6) in DALYs due to low birthweight.

DALYs due to late-onset neonatal sepsis caused by short gestation and low birthweight in both sexes in the USA initially increased and subsequently decreased, indicating a slight overall upward trend. In contrast, a stable decreasing trend in the DALYs due to short gestation and low birthweight was observed in China. Male neonates with late-stage neonatal sepsis in China had the highest decreasing trend values between the two age groups stratified by country; however, a slightly increasing trend in DALYs due to short gestation (APC: −1.7; 95% CI: −2.0, −1.4) and low birthweight (APC: −2.3; 95% CI: −2.7, −2.0) was observed in both groups between 2013 to 2019. Generally, the DALYs due to neonatal sepsis caused by the two aforementioned risk factors were higher in neonates with early-onset neonatal sepsis than in those with late-onset in both countries.

### Temporal trends in the SEVs for short gestation and low birthweight, 1990–2019

**Figure 4.**
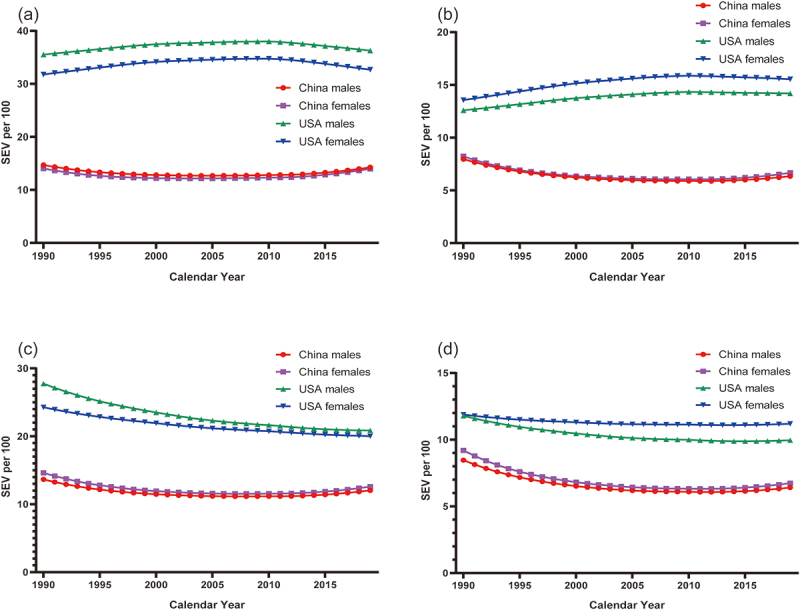
The temporal trends in the age-standardized SEV per 100 population of short gestation and low birth weight in early- and late-neonates by sex in China and the USA, 1990–2019. (a). SEV of short gestation in early neonates. (b). SEV of low birth weight in early neonates. (c). SEV of short gestation in late neonates. (d). SEV of low birth weight in late neonates.

Abbreviation: SEV summary exposure value

The curves of SEVs for short gestation and low birthweight are shown in Figure 4. Overall, the SEVs for both short gestation and low birthweight in neonates with early- and late-onset neonatal sepsis were lower in China than in the USA, with both initially exhibiting a decrease, followed by a slight increase. Conversely, in the USA, the SEVs for the two aforementioned risk factors initially increased and subsequently decreased in neonates with early-onset neonatal sepsis while exhibiting a consistent decrease in those with late-onset neonatal sepsis. The magnitude of change in all of the aforementioned trends was small.

Overall, the temporal trend changes in the mortality rate of neonatal sepsis derived from the joinpoint regression models were the same as the temporal trend changes in YLLs. The mortality rates were not separately tabulated and plotted.

## Discussion

Overall, the results of the present study revealed an increasing trend in the incidence and prevalence of early- and late-onset neonatal sepsis in China between 1990 and 2019 compared with that in the USA, which is consistent with the findings of Tao Pan et al. [31]. In addition, a decreasing trend in DALYs due to neonatal sepsis was observed in both China and the USA, and a sustained decline in DALYs due to short gestation and low birthweight was observed in China, possibly due to the better management of risk factors for neonatal sepsis. The incidence of sepsis and DALYs due to sepsis was higher among neonates with early-onset neonatal sepsis than among those with late-onset neonatal sepsis. YLDs accounted for only a small percentage of DALYs; however, a significant upward trend in YLDs was observed in China. This may be due to several reasons. First, the survival rate of neonates with short gestation and low birthweight might have increased following the advancements of neonatal speciality care medicine. However, many surviving infants cannot be fully rehabilitated, resulting in more persistent disabilities [32]. Second, many neonates in China are discharged early against medical advice and without comprehensive care due to the heavy financial burden on the child’s family caused by the lack of comprehensive health insurance coverage [33]. This reduces the survival rate of neonates with short gestation and low birthweight and increases the risk of complications such as cerebral palsy. Therefore, the aim of the Chinese public health policy should be to gradually promote comprehensive health insurance coverage, improve the social welfare system, develop a perinatal regionalisation system, and enhance neonatal follow-up and developmental care [34]. Third, neonatal sepsis may lead to cerebral white matter damage and increase the susceptibility of the brain to subsequent injury through a free radical attack, the production of pro-inflammatory cytokines, and hypoxic-ischemic encephalopathy caused by hypotension and impaired autoregulation of cerebral blood flow [35]. Following the recent development of measures to assess and document neurocognitive outcomes, long-term follow-up of high-risk populations has intensified, and the outcomes of disability due to neonatal sepsis have been further investigated [36]. Enhancing sensory stimulation and other measures may help in neonatal brain development, thereby mitigating neurological deficits [37].

Short gestation and low birthweight are the two critical risk factors for the development of neonatal sepsis [38,39]. Overall, the incidence of neonatal sepsis is inversely associated with gestational age, with 33% of infants born at <28 weeks and approximately 60% of those born at <25 weeks ultimately developing neonatal sepsis [39]. Furthermore, preterm infants with low birthweight are 3–10 times more likely to develop sepsis than are full-term infants with normal birthweight [12]. Low immunoglobulin G levels also predispose infants with short gestation and low birthweight to infection because of a reduced activation of the complement system, which promotes antibody-dependent cytotoxicity and opsonisation [40]. Notably, the rapid dominance of Amoeba species in the gut microbiota of such neonates during the first week of life, which remains at high levels until 28 days after birth, also induces low immunity. These infants also have a high risk of requiring invasive mechanical ventilation and long hospital stays, resulting in an increased risk of hospital-acquired infections and a high incidence of necrotising small-bowel colitis, which can further result in neonatal sepsis [30,31]. The results of the present study revealed that, in China, the SEVs of neonates with short gestation and low birthweight decreased and subsequently increased in cases of early- and late-onset neonatal sepsis. This initial decline is associated with measures such as universal preconception and pregnancy care, early detection of pregnancy complications, and timely interventions [34]. In recent decades, the rapid economic development of China has boosted the development of healthcare system. For instance, a substantial development was achieved in 2013 when the Chinese government implemented a risk management strategy to reduce the maternal mortality rate. This strategy involved establishing a national referral and treatment network for critically ill mothers and newborns, with 3369 and 3070 maternal and neonatal intensive care centres established, respectively [41]. Neonates with low birthweight have a poor ability to pump and reserve milk, possibly leading to hypoglycaemia, which is one of the reasons underlying the high mortality rate of neonatal sepsis [42]. Therefore, enhanced monitoring of neonatal blood glucose and timely correction of hypoglycaemia are crucial to reducing the rate of mortality due to neonatal sepsis. In addition, increased training of families and health workers in specialised neonatal sepsis awareness to improve the recognition of the early stages of infection is crucial to achieving sufficient time for treatment [43].

This close relationship between economic development and healthcare improvement is understandable, considering the gross domestic product negatively correlates with infant mortality (in the long term) and positively correlated with life expectancy [44]. However, several difficulties in perinatal management in China should be considered. First, the two-child policy has significantly increased the proportion of pregnant women of advanced age, multipara, and irregular prenatal checkups, with a corresponding increase in the proportion of pregnant women at high risk for complications [45]. Second, since 2018, national regulations have indicated that medical expenses for rural women giving birth in the hospital will only be paid using the basic medical insurance for urban and rural residents and that medical expenses for childbirth will no longer be subsidised by the Chinese government [41]. Providing quality health care to pregnant women is necessary to prevent premature birth; thus, this policy is likely to adversely impact neonatal care [46]. Third, in 2019, China still had the third highest incidence of preterm birth in the world, placed after only India and Pakistan [19]. Finally, improving rates of recovery from primary infections and reducing sequelae due to end-organ damage through improvements in treatment practices remain crucial to reducing the disease burden of neonatal sepsis in China. Therefore, China needs to learn further from the perinatal management practices and experiences of high-income countries to improve its neonatal network and care techniques, optimise obstetrics and drug treatment, and improve health services [33]. Examples of such initiatives include ensuring that all births are attended by skilled staff whenever possible and increasing coverage of prenatal steroid treatment [19]. It is also vital to improve hand hygiene, use less invasive care, improve breastfeeding rates, and provide developmental care for sepsis in preterm infants [47]. Various risk stratification and scoring systems conducted in the USA may help determine whether to initiate empiric antibiotic therapy after birth. Notably, these systems not only have helped to standardise the decision to begin treatment but have also reduced the rate of antibiotic use by 75% [48]. Furthermore, poverty and lack of education among women are reported associated with the disease burden of neonatal sepsis [49]. The provision of economic and educational assistance to mothers, such as increased government grants or public funds, may be a useful initiative.

This study has some limitations. First, owing to the limited availability and quality of raw data in the GBD 2019 and the need to further improve in the standardisation of data collection and measurement methods, the accuracy and stability of estimates might have been compromised [3]. Second, the definition of neonatal sepsis may be modified in low- and middle-income countries and regions where laboratory testing is unavailable, leading to mis-classification and miscoding and thus affecting the accuracy of the raw data. Finally, the combined role of risk factors was not considered, which might have compromised estimates of the risk-attributed disease burden.

## Conclusions

The aim of the Chinese public health policy should be to control the risk factors for short gestation and low birthweight, further develop neonatal specialty care medicine, promote effective obstetric techniques, enhance perinatal care, and improve neonatal follow-up and rehabilitation interventions.

## Supplementary Material

Supplementary table 3_.docx

Supplementary table 1.docx

Supplementary table 2.docx

## Data Availability

Public information is available online (http://ghdx.healthdata.org/gbd-results-tool)

## List of abbreviations

GBD: Global Burden of Disease Study
USA: United States of America
DALYs: Disability-adjusted life years
CODEm: Cause of Death Ensemble Model
YLLs: Years of life lost
YLDs: Years lived with disability
SEV: Summary exposure value
GATHER: Guidelines for Accurate and Transparent Health Estimates Reporting
APC: Annual percentage change
AAPC: Average annual percentage change
MMR: Maternal mortality ratio

## Data deposition

The dataset supporting the conclusions of this article is available in the DANS EASY database: Zhang, C.Y. (Zhejiang University School of Medicine) (2023): Epidemiological data on neonatal sepsis in China and the United States. DANS. https://doi.org/10.17026/dans-z4m-bfwe.

## Preprints

This manuscript has been posted as a preprint on Research Square with doi: https://doi.org/10.21203/rs.3.rs-3132346/v1, which is available from: https://www.researchsquare.com/article/rs-3132346/v1.
