# Supplementary material for: Disease burden comparison and associated risk factors of early- and late-onset neonatal sepsis in China and the USA, 1990–2019
Source: Glob Health Action. 2024 Sep 4;17(1):2396734. doi: 10.1080/16549716.2024.2396734 (PMC11376289; doi:10.1080/16549716.2024.2396734)
Supplement: Supplementary table 3_.docx [file ZGHA_A_2396734_SM2769.docx]

**Supplementary table 3.**

Prevalence, incidence, DALYs, YLDs and YLLs of early and late onset neonatal sepsis by sex in China and the USA, 1990 - 2019

|  | Prevalence | | Incidence | | DALYs | | YLDs | | YLLs | |
| --- | --- | --- | --- | --- | --- | --- | --- | --- | --- | --- |
|  | Early  neonates | Late neonates | Early  neonates | Late neonates | Early  neonates | Late neonates | Early  neonates | Late neonates | Early  neonates | Late neonates |
| 1990 | | | | | | | | | | |
| China males | 1094.09 | 1081.68 | 144902.33 | 26768.75 | 46724.52 | 14528.13 | 83.17 | 80.57 | 46641.35 | 14447.56 |
| China females | 1005.64 | 997.86 | 127816.28 | 27379.57 | 26795.26 | 9877.68 | 65.28 | 63.04 | 26729.98 | 9814.64 |
| USA males | 333.16 | 332.60 | 43832.88 | 8305.63 | 63382.80 | 15048.79 | 15.31 | 15.39 | 63367.48 | 15033.40 |
| USA females | 281.55 | 281.23 | 35646.49 | 7661.79 | 49675.56 | 12306.82 | 10.14 | 10.23 | 49665.43 | 12296.59 |
| 2019 | | | | | | | | | | |
| China males | 1972.86 | 1964.08 | 268886.08 | 31594.36 | 27715.45 | 7313.00 | 128.13 | 126.24 | 27587.32 | 7186.77 |
| China females | 2032.79 | 2024.69 | 273306.10 | 32516.62 | 19704.76 | 6125.18 | 138.15 | 135.95 | 19566.61 | 5989.23 |
| USA males | 334.67 | 334.32 | 38418.65 | 9556.64 | 38153.06 | 14203.19 | 6.82 | 6.81 | 38146.25 | 14196.39 |
| USA females | 279.42 | 279.16 | 31749.75 | 8151.07 | 32493.10 | 12221.97 | 5.25 | 5.24 | 32487.85 | 12216.73 |

^*^ Significantly different from 0 (P < 0.05).

*Abbreviation:* DALYs disability-adjusted life years; YLDs years lived with disability; YLLs years of life lost.
