# Supplementary material for: Disease burden comparison and associated risk factors of early- and late-onset neonatal sepsis in China and the USA, 1990–2019
Source: Glob Health Action. 2024 Sep 4;17(1):2396734. doi: 10.1080/16549716.2024.2396734 (PMC11376289; doi:10.1080/16549716.2024.2396734)
Supplement: Supplementary table 2.docx [file ZGHA_A_2396734_SM2767.docx]

**Supplementary table 2.**

Trends in prevalence and incidence of late onset neonatal sepsis by sex in China and the USA, 1990 - 2019, using Joinpoint regression models

|  | Prevalence | | Incidence | |
| --- | --- | --- | --- | --- |
|  | Time interval | APC (95% CI) | Time interval | APC (95% CI) |
| China males | | | | |
| Trend 1 | 1990-1995 | 0.1 (-0.3, 0.5) | 1990-2000 | -1.0 (-1.2, -0.8) ^*^ |
| Trend 2 | 1995-2011 | 3.0 (3.0, 3.1)^*^ | 2000-2005 | 3.8 (3.0, 4.7)^*^ |
| Trend 3 | 2011-2017 | 1.1 (0.7, 1.4)^*^ | 2005-2009 | -2.5 (3.7, -1.2) ^*^ |
| Trend 4 | 2017-2019 | 2.6 (1.0, 4.3)^*^ | 2009-2019 | 1.7 (1.5, 1.9)^*^ |
| AAPC | 1990-2019 | 2.1 (1.9, 2.2) ^*^ | 1990-2019 | 0.6 (0.3, 0.8) ^*^ |
| China females | | | | |
| Trend 1 | 1990-1996 | 0.4 (-0.2, 1.0) | 1990-1994 | -1.5 (-2.0, -0.9)^*^ |
| Trend 2 | 1996-2003 | 3.0 (2.4, 3.6)^*^ | 1994-2000 | -0.6 (-1.0, -0.2)^*^ |
| Trend 3 | 2003-2010 | 4.6 (4.0, 5.2)^*^ | 2000-2017 | 0.9 (0.9, 1.0)^*^ |
| Trend 4 | 2010-2019 | 1.6 (1.3, 1.9)^*^ | 2017-2019 | 5.1 (3.2, 7.0)^*^ |
| AAPC | 1990-2019 | 2.4 (2.1, 2.6)^*^ | 1990-2019 | 0.6 (0.4, 0.7)^*^ |
| USA males | | | | |
| Trend 1 | 1990-1995 | 0.9 (0.7, 1.2)^*^ | 1990-1997 | 0.7 (0.6, 0.8)^*^ |
| Trend 2 | 1995-2000 | -1.5 (-1.9, -1.2) ^*^ | 1997-2009 | 1.5 (1.4, 1.5)^*^ |
| Trend 3 | 2000-2008 | 1.3 (1.2, 1.5)^*^ | 2009-2017 | -0.5 (-0.5, -0.4)^*^ |
| Trend 4 | 2008-2019 | -0.6 (-0.7, -0.5)^*^ | 2017-2019 | -2.4 (-3.1, -1.8)^*^ |
| AAPC | 1990-2019 | 0.0 (-0.1, 0.1) | 1990-2019 | 0.5 (0.4, 0.6)^*^ |
| USA females | | | | |
| Trend 1 | 1990-1995 | 0.8 (0.6, 1.0)^*^ | 1990-2001 | 0.1 (-0.1, 0.2) |
| Trend 2 | 1995-2000 | -1.6 (-1.9, -1.3)^*^ | 2001-2010 | 1.1 (0.9, 1.3)^*^ |
| Trend 3 | 2000-2009 | 0.8 (0.7, 0.9)^*^ | 2010-2016 | -0.1 (-0.5, 0.4) |
| Trend 4 | 2009-2019 | -0.4 (-0.4, -0.3)^*^ | 2016-2019 | -1.8 (-2.7, -0.9)^*^ |
| AAPC | 1990-2019 | -0.0 (-0.1, 0.1) | 1990-2019 | 0.2 (0.0, 0.3)^*^ |

^*^ Significantly different from 0 (P < 0.05).

*Abbreviation:* APC annual percent change; AAPC average annual percent change; CI confidential interval
